# Utility of Unrefined Carbohydrates in Type 2 Diabetes. Comment on “Reversing Type 2 Diabetes: A Narrative Review of the Evidence, *Nutrients*, 2019, *11*, 766”

**DOI:** 10.3390/nu11071620

**Published:** 2019-07-17

**Authors:** Shivam Joshi, Timothy Zaki, Robert J. Ostfeld, Michelle McMacken

**Affiliations:** 1 Division of General Internal Medicine, Department of Medicine, New York University School of Medicine, New York, NY 10016, USA; 2 Department of Medicine, NYC Health + Hospitals/Bellevue, New York, NY 10016, USA; 3 New York University School of Medicine, New York, NY 10016, USA; 4 Division of Cardiology, Montefiore Health System, Bronx, NY 10467, USA

Hallberg et al. provide a limited literature review on the reversal of type 2 diabetes mellitus (T2DM) [1]. Insulin resistance—not carbohydrate excess—is at the root of T2DM. Indeed, Anderson et al. fed 20 participants with T2DM a diet high in fiber and unrefined carbohydrates (approximately 70% of calories) and demonstrated short-term reversal through the discontinuation of all T2DM treatment in 11 patients over an average of 16 days; notably, this reversal was accomplished in the absence of weight loss [2]. In contrast, Rosenbaum et al. recently showed that four weeks of a ketogenic diet resulted in higher post-prandial serum glucose values and lower insulin sensitivity in response to a control meal, compared with four weeks of a non-ketogenic diet [3].

Another critique of the Hallberg et al. review is the omission of important limitations in the study by Saslow et al. [4]. In the 34-week trial by Saslow et al., researchers demonstrated the short-term “reversal” of T2DM in 55% of patients in the ketogenic arm compared to 0% in the low-fat arm. However, compared to the low-fat arm, the ketogenic arm received more than double the number of “lessons” with their diet, which also included “the importance of physical activity and sleep” and “positive affect regulation and mindful eating materials”; these confounding variables suggest that any improvement in diabetic parameters cannot be solely ascribed to the change in diet. Another study cited by Hallberg et al. also exhibited pronounced differences in behavioral support between the two dietary arms, which, along with the self-selection and non-randomization of patients, may have contributed to an effect [5].

Regarding another study, Hallberg et al. describe an impressive 78% “reversal” rate of diabetes at one year using a definition that excludes metformin [6]; however, this seems counterintuitive given that metformin—a medication whose impact on HbA1c in T2DM is well-recognized—is a mainstay of T2DM treatment. It is also important to note that the mean baseline HbA1c in the intervention arm was 6.6%, barely above the target HbA1c used for reversal and lower than the control arm’s mean of 6.9%. After one year, the net benefit of the intervention arm over the control arm was only a 0.3% reduction in HbA1c, suggesting that the short-term benefits of very-low carbohydrate diets (VLCDs) likely wane with time. In fact, four randomized, long-term (≥12 months) trials of VLCDs for T2DM have failed to show a benefit over control (typically high-carbohydrate) diets [7,8,9,10]. Notably, none of these four studies were conducted in association with Virta Health, a fee-based VLCD program with which 75% of the Hallberg et al. authors have disclosed a financial conflict of interest. 

Ultimately, the review by Hallberg et al. presents an overly enthusiastic narrative – and not systematic – review of VLCDs for the treatment of T2DM that does not reflect the entirety of the current evidence available. The authors omit a discussion of high-carbohydrate diets being used to treat and reverse T2DM and fail to mention key limitations of the studies cited on ketogenic diets. Moreover, the authors’ working definition of “reversal” does not necessarily reflect improvements in the underlying pathophysiology of T2DM, i.e., insulin resistance or carbohydrate intolerance.
